# Utilizing Solvent Repulsion between Dimethylformamide and Isopropanol to Manipulate Sn Distribution for Bifacial Cu_2_ZnSn(S,Se)_4_ Solar Cells

**DOI:** 10.1021/acsaem.4c01905

**Published:** 2024-11-27

**Authors:** Alice Sheppard, Raphael Agbenyeke, Jude Laverock, Laurence King, Jacques Kenyon, Nada Benhaddou, Nicole Fleck, Robert L. Harniman, Andrei Sarua, Devendra Tiwari, Jake W. Bowers, Neil A. Fox, David J. Fermin

**Affiliations:** † School of Chemistry, University of Bristol, Cantocks Close, BS8 1TS Bristol, U.K.; ‡ H. H. Wills Physics Laboratory, University of Bristol, Tyndall Avenue, BS8 1TL Bristol, U.K.; § Centre for Renewable Energy Systems Technology (CREST), Wolfson School of Mechanical Electrical and Manufacturing Engineering, Loughborough University, LE11 3TU Loughborough, U.K.; ∥ Department of Mathematics, Physics and Electrical Engineering, Northumbria University, Ellison Place, NE1 8ST Newcastle Upon Tyne, U.K.

**Keywords:** Cu_2_ZnSn(S,Se)_4_, thin-film PV, solution processing, Sn disorder, photoemission of electrons microscopy

## Abstract

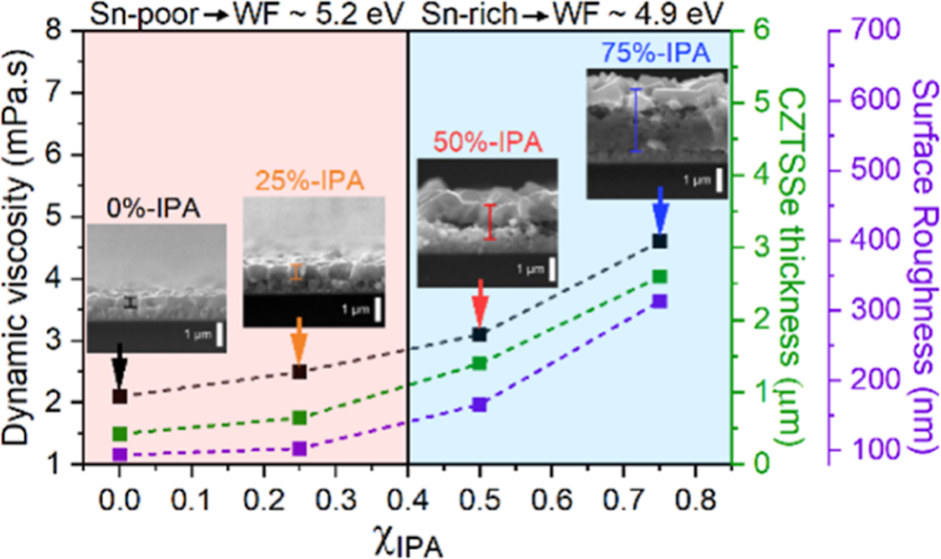

Rationalizing the role of chemical interactions in the precursor solutions on the structure, morphology, and performance of thin-film Cu_2_ZnSn(S,Se)_4_ (CZTSSe) is key for the development of bifacial and other photovoltaic (PV) device architectures designed by scalable solution-based methods. In this study, we uncover the impact of dimethylformamide (DMF) and isopropanol (IPA) solvent mixtures on cation complexation and rheology of the precursor solution, as well as the corresponding morphology, composition, and PV performance of CZTSSe thin-film grown on fluorine-doped tin oxide (FTO). We find that increasing the proportion of IPA leads to a nonlinear increase in dynamic viscosity due to the strong repulsion between DMF and IPA, which is characterized by an interaction cohesion parameter of 3.06. The repulsive solvent interaction not only leads to complex dependence on absorber thickness and surface roughness but also on composition disorder in the annealed CZTSSe films. Systematic studies involving Raman, scanning electron microscopy, SIMS, XPS, and energy-filtered photoemission of electron microscopy show that adding 25% of IPA to DMF leads to thin films with a high degree of structure and composition homogeneity in comparison to pure DMF-based precursors. Further increasing the IPA content promotes Sn surface segregation and secondary phases, which have a clear impact on the surface electronic landscape of the absorber layer. This analysis allows for the rationalization of the device performance with the stack configuration glass/F:SnO_2_/CZTSSe/CdS (50 nm)/i-ZnO (50 nm)/Al:ZnO (500 nm)/Ag (500 nm).

## 1 Introduction

Building integrated photovoltaics (BIPVs), such as bifacial, semitransparent, or flexible alternatives, is among the fastest growing sectors within PV and provides a way to lower the carbon footprint from power generation in densely populated regions.^1^ Thin-film PV technologies based on materials such as Cu(In,Ga)Se_2_ and Cu_2_ZnSn(S,Se)_4_ (CZTSSe) offer unique advantages over Si for BIPV technologies, such as lightweight, flexible, and bifacial modules.^2−5^

With recent breakthroughs using solution processing and defect-suppressing elemental substitutions, power conversion efficiencies (PCEs) are approaching 15%^6−10^ for CZTSSe on conventional Mo-coated glass substrates, reinvigorating interest in this research area.^6−10^ Successful kesterite devices deposited on tin-doped indium oxide (ITO) and fluorine-doped tin oxide (FTO) transparent conductive oxides (TCOs) have been reported in literature.^11−15^ However, an interfacial reaction between ITO and CZTSSe was found to occur during a 500 °C selenization, leading to In diffusion into CZTSSe and the formation of SnO_2_, degrading the conductivity of the back contact.^13,14^ Kim et al.^16^ fabricated CZTSe on an FTO substrate, achieving a PCE of 6.05% under bifacial illumination, finding that no interfacial reaction occurs between CZTSe and FTO, indicating that FTO is more suitable for bifacial CZTSSe applications.^17,18^ Strategies to boost CZTSSe PCEs on FTO up to 11.4% have included depositing Mo:Na or MoO_3_ nanoscale layers,^19,20^ as well as transition-metal oxides, such as TiO_2_ and V_2_O_5_.^21,22^ However, little attention has been paid to the composition of the molecular precursor ink and how this affects the morphology and electronic properties of the absorber layer.

Solution processing of CZTSSe thin-films at the laboratory scale commonly involves the spin coating of molecular precursor inks, formulated by dissolving metal salts with a chalcogen (S or Se) in a chosen solvent. Aprotic solvents, such as dimethylformamide (DMF) and dimethyl sulfoxide (DMSO), have been a widely used solvent for these applications, with PCEs of up to 13% reported on Mo.^6,10,23−26^ There have also been reports of using a binary DMF/DMSO solvent blend for CZTSSe devices,^27−29^ as well as the addition of small amounts of protic solvents, such as water and acetone, for DMSO and 2-methoxyethanol (2-MOE)-based kesterite precursor inks.^30−32^ An equivolume of DMF and isopropanol (IPA) was used for our previous CZTS work,^33−35^ however, no systematic studies have been reported on the impact of blending an aprotic and protic solvent on the structure, composition, and optoelectronic properties of CZTSSe thin-film absorbers.

In this study, we uncover, for the first time, the complex interaction generated by DMF/IPA solvent blends on the precursor solution rheology and their impact on grain growth, morphology, elemental disorder, and optoelectronic properties of CZTSSe thin films. The strong solvent repulsion between DMF and IPA results in a nonlinear increase of dynamic viscosity of the precursor solution, CZTSSe film thickness, and surface roughness, upon increasing the IPA content from 0 to 75%. Furthermore, the IPA content has a strong effect on Sn disorder across the film, which has a profound impact on the surface electronic landscape as probed by energy-filtered photoemission of electron microscopy (EF-PEEM). We correlate these properties with PV device performance on grown optically transparent F:SnO_2_ surfaces.

## 2 Results and Discussion

### 2.1 Properties of DMF/IPA Solvent Blends

**Figure 1:**
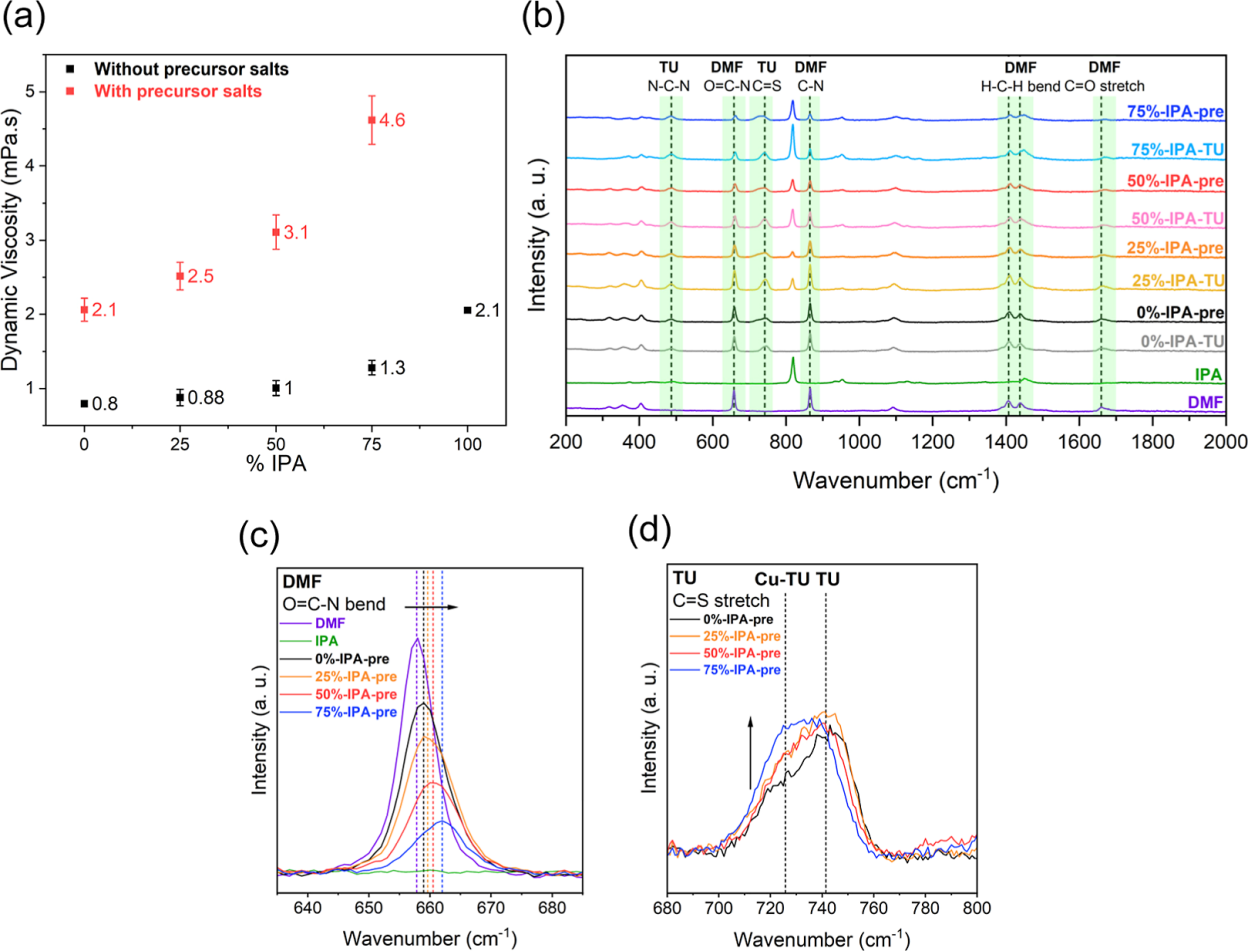
Dynamic viscosity measurements of the precursor solution in the presence and absence of metal precursors and thiourea at 25 °C (a). Details of the precursor composition can be found in the Supporting Information S1. The dynamic viscosities quoted for pure DMF and IPA without salts were obtained from literature.^36,37^ Raman spectra of DMF solution with various IPA content in with or without thiourea TU (*X* %-IPA-TU) and metal cations (*X* %-IPA-pre) from 200 to 2000 cm^–1^ (b), with a magnified image of DMF O=C–N DMF bending (c) and TU C=S stretching vibrations (d). *X* corresponds to the percentage of IPA in the precursor solution.

Figure 1a shows the nonlinear increase in dynamic viscosity of DMF and IPA solvent blends, measured using a reverse flow U-tube viscometer (details provided in the Supporting Information S1), rising from 0.8 to 1.3 mPa s upon increasing the IPA ratio from 0 to 75%. The physical properties of pure DMF and IPA solvents are summarized in Table S1.^36,37^ This nonlinear relationship in dynamic viscosity is found to follow the Lederer–Roegiers equation for solvent blends (Figure S1).^38−40^ By fitting with eq S1, the interaction cohesion parameter (α) between DMF and IPA was measured to be 3.06, which indicates strong separation between the solvents in the mixture.^41^ Upon the addition of the precursor salts, there is clear increase in viscosity, which is more significant at higher IPA content. This observation can be attributed to the greater separation between DMF and IPA upon the dissolution of precursor salts in DMF, increasing fluid friction between the solvent phases.

Raman spectroscopy (Figure 1b–d) shows a clear change in bond length of the DMF O=C–N bending (∼660 cm^–1^) and TU C=S stretching (∼740 cm^–1^) modes with varying the proportion of IPA in the precursor solution, indicating an interplay of effects due to changing dielectric constant of the medium, changing intermolecular interaction, and precursor complexation. In the case of the precursor solutions, IPA diffusion into the DMF environment weakens the DMF intermolecular effects (H-bonding for the DMF O=C stretch) and leads to a shift to higher wavenumbers and peak broadening, revealing the emergence of new solvent–solute interactions with increasing proportions of IPA in the solution (Figures 1c and S2). According to Figure 1b, there are no detectable shifts in the N–C–N (486 cm^–1^) bending mode for TU or C–N (865 cm^–1^), and H–C–H bending (1407 and 1438 cm^–1^) modes of DMF, suggesting no detectable interaction with any of these functional groups.

Upon the addition of the Cu, Sn, and Zn salts to the solution, a shoulder peak emerges at TU in the region of C=S stretching mode around 727 cm^–1^ (Figure 1d), which can be associated with the formation of CuCl_*x*_-TU coordination and the disappearance of the double bond character of the C=S upon complexation.^42,43^ Our recent studies have shown that TU metal complexes self-assemble into submicron size colloidal species which play an important role on the morphology and optoelectronic properties of CZTSSe films.^44^ The relative intensity of the peak associated with coordinated Cu-TU at 727 cm^–1^ to uncoordinated TU at 740 cm^–1^ increases with a higher proportion of IPA in the CZTS precursor ink, indicating a greater Cu-TU complexation.

### 2.2 From Precursor Solutions to CZTSSe Absorbers

**Figure 2:**
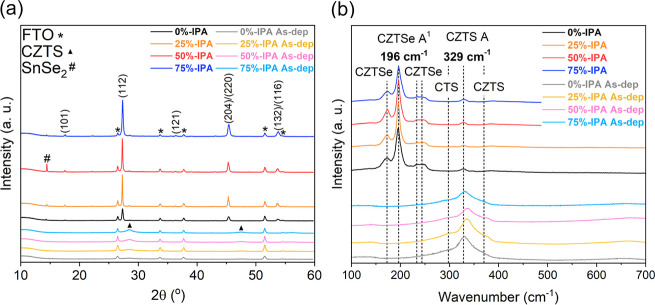
XRD (a) and Raman (b) spectra under 532 nm laser excitation of the as-deposited dry precursor (As-dep) and after reactive annealing of thin-films absorber deposited on FTO from precursor solution with various solvent blends.

Figure 2 shows the evolution of XRD and Raman spectra of the thin films, obtained from the various solvent mixtures, before and after reactive annealing on FTO substrates. Diffraction patterns consistent with polycrystalline CZTSSe are observed upon selenization, including the (112), (204)/(220), and (132)/(116) planes of CZTSSe (JCPDS 04-019-1847) at approximately 27.3, 45.3, and 53.7°, respectively. By analyzing the (112) diffraction peak position (as described in the Experimental Details),^45^ a S/(S + Se) chalcogenide ratio of 0.3 was estimated for all CZTSSe absorbers, confirming a high degree of S replacement with Se for all films. As summarized in Table S2, a significant decrease in full width at half-maximum (fwhm) of the XRD features is observed for 25%-IPA, which then increases upon further addition of IPA. Additionally, SnSe_2_ can be identified in the XRD spectra for 50%-IPA, with the (001) plane at the diffraction angle of 14.5° (JCPDS 01-089-2939). This observation reveals a surprising nonmonotonic change in crystalline size domains and secondary phase nucleation patterns as a function the solvent blend at the molecular precursor level.

A similar trend is observed with the fwhm of the A^1^ vibrational mode obtained from Raman spectroscopy, as also shown in Table S2. Figure 2b shows clear evidence of the Cu_2_Sn(S,Se)_3_ (CTS) phase, with a shoulder peak at 299 cm^–1^,^46^ in the precursor layer (As-dep), obtained due to heating the substrate at 350 °C after each spin-coating step. However, after the reactive annealing step, the Raman spectra primarily feature the vibrational modes of CZTSSe.^47,48^ These results suggest that the formation of CZTSSe follows the thermal conversion of CTS, regardless of the solvent composition of the molecular precursor.

**Figure 3:**
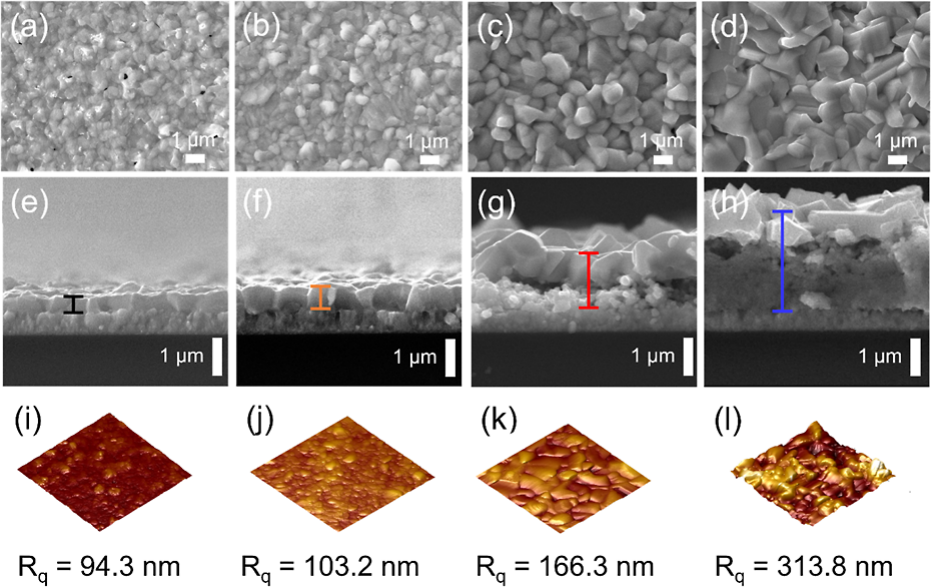
Top-down (a–d) and cross sectional (e–h) SEM images, as well as 10 × 10 μm^2^ AFM images of annealed 0%-(a,e,i), 25%-(b,f,j), 50%-(c,g,k), and 75%-IPA (d,h,l) CZTSSe films on FTO.

SEM and AFM images, as shown in Figure 3, illustrate the impact of DMF and IPA ratio in the molecular precursor ink on the nucleation and morphology of the annealed CZTSSe films. 0%-IPA has small CZTSSe grains with pinholes and a film thickness of 0.43 μm after 13 spin coating and drying steps (Figure 3a,e). Introducing 25% of IPA improves grain size and thickness (0.65 μm) when comparing IPA free precursors. Further increasing the IPA content up to 75% leads to larger grain sizes and thicker films of 1.4 (Figure 3g) and 2.6 μm (Figure 3h), respectively, with a lower grain packing density. Analysis of the AFM images in Figure 3i–l (further images are shown in Figure S3) reveals a nonlinear increase of the surface roughness (*R*_q_) with increasing proportion of IPA in the precursor ink. These results clearly illustrate the close correlation between dynamic viscosity of the precursor solution with film thickness and surface roughness of the annealed CZTSSe film. 75%-IPA CZTSSe exhibits a bilayer structure with a thickness of 2.6 μm (Figure 3h), comprising a large-grain, loosely packed top layer, and a fine-grain bottom layer. Secondary ion mass spectroscopy (SIMS) analysis in Figure S4 reveals that the fine-grain layer is carbon- and Zn-rich (Cu/Zn ∼ 1), which is absent in the case of 25%-IPA. The high carbon content in 75%-IPA CZTSSe films is likely to originate from decomposition of IPA during the multiple drying steps and subsequent reactive annealing to form the CZTSSe phase.

Figures S3a,d illustrate a larger scale topographic analysis of the films, in which SnSe_2_ elliptical crystals can be observed at the surface of 50%-IPA (Figure S3c). The composition of these crystals was confirmed by EDX mapping (Figure S3i). As we discussed further below, these phases have an impact on the device performance. Interestingly, this observation is consistent with the XRD features, as shown in Figure 2a. Based on this unprecedented observation, we propose that the molecular boundary between poorly mixing DMF and IPA acts as nucleation sites for the Sn phases, potential SnS_2_, which are readily selenized during the annealing process. We shall discuss further evidence of solvent-induced Sn disorder in the next section.

### 2.3 Effect of DMF and IPA Solvent Repulsion on Sn Distribution

**Figure 4:**
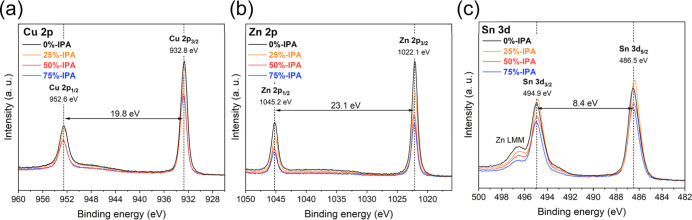
XPS spectra of Cu 2p (a), Zn 2p (b), and Sn 3d (c) for CZTSSe absorber films, processed with the various solvent blends. Details of surface pretreatment are described in the Supporting Information S1.

Figure 4 shows the photoemission spectra of Cu 2p, Zn 2p, and Sn 3d in CZTSSe films as a function of the IPA content in the precursor solution. These spectra were recorded after a judicious surface pretreatment, as detailed in Figure S5. For Cu 2p and Zn 2p, the satellite spectra are consistent with Cu^+^ and Zn^2+^.^49,50^ The binding energies of the main Sn 3d peaks are consistent with those of Sn^4+^. The Sn 3d_5/2_ peak in Figure 4c also shows some broadening, which can be associated with additional Sn phases. As described in Figure S6 and Table 1, we have estimated the percentage of Sn^4+^ in secondary phases, which increases from 20 to 33% upon increasing IPA content up 50%, decreasing back to 26% in 75%-IPA. SEM analysis (Figure S3) reveals that SnSe_2_ coverage is far lower than 20–33%, indicating that additional Sn environments, such as Sn_Zn_ defects, are likely present.^51,52^

**Table 1:** Surface Cation Compositions of Annealed CZTSSe Absorber Surfaces from XPS^a^

	surface CZTSSe composition				
% IPA	Cu/(Zn + Sn)	Zn/Sn	ratio of Sn^4+^ phases-XPS (%)	WF–EF–PEEM (eV)	WF distribution fwhm (eV)
0	0.68	1.57	21.0	5.18	115
25	0.75	1.09	20.5	5.25	71
50	0.73	0.93	33.0	4.87	135
75	0.79	0.89	25.9	4.85	107

a Sn amount includes both Sn contributions.

As summarized in Table 1, the surface Cu/(Zn + Sn) ratio is slightly lower in the case of 0%-IPA, in comparison to the other solvent formulations. On the other hand, Zn/Sn ratio decreases from 1.57 to 0.89 upon increasing the IPA content in the precursor from 0 to 75%. These data show that as the amount of IPA increases, the surface composition changes from Zn-rich to Sn-rich, with 25%-IPA showing the surface composition closer to the precursor ink (bulk) composition. This trend is also confirmed by SIMS analysis in Figure S4a, revealing a uniform elemental composition for the 25%-IPA CZTSSe film.

Another interesting observation is the approximately 0.1 eV upward shift of the valence band maximum (VBM) in the case of 25%-IPA, as estimated from the ultraviolet photoemission spectra in Figure S7. This is consistent with the 0.1 eV shift of the Cu 2p, Zn 2p, and Sn 3d photoemission lines in the XPS data observed in 25%-IPA films. This set of observations provides clear evidence that the mixture of solvents in the precursor solution has significant impact on Sn disorder across the CZTSSe film.

**Figure 5:**
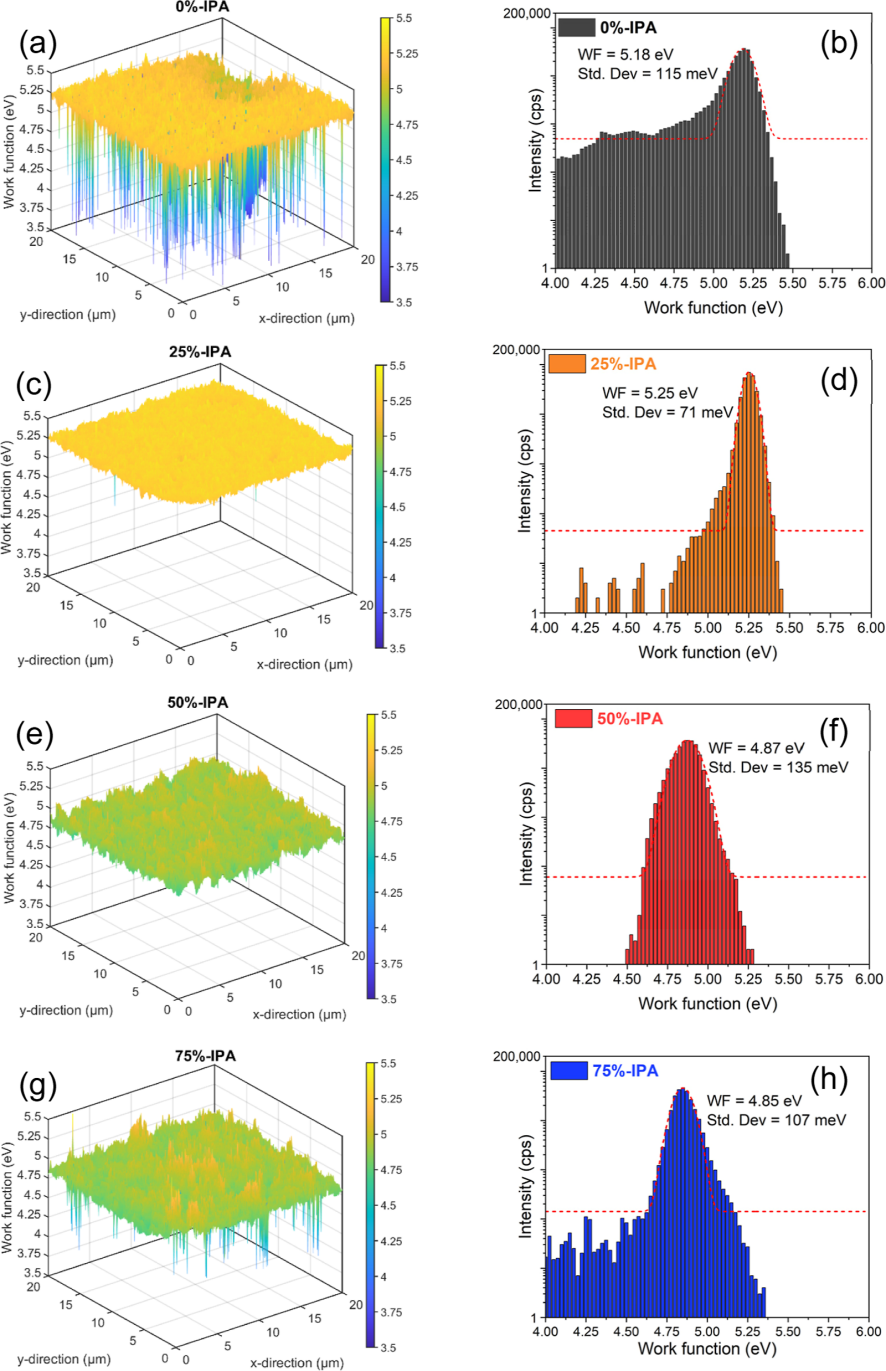
Energy-filtered photoemission of electrons microscopy. Local work function maps (a,c,e,g) and the corresponding work function histogram (b,d,f,h) on a 20 μm field of view, showing the impact of the solvent blend in the surface electronic landscape of CZTSSe.

Figure 5 displays the spatially resolved 20 μm × 20 μm work function (WF) maps of CZTSSe films probed by energy-filtered photoemission electron microscopy (EF-PEEM) under monochromatic He I (21.22 eV) illumination. It is important to note that the direct EF-PEEM value of WF, determined from the low-energy emission threshold,^53^ is dependent on surface electron density, topography, and local electrostatic fields.^54^ Two parameters can be extracted from these data, the center of the WF distribution and the standard deviation (Std. Dev.) of the fitted Gaussian curve, as reported in Table 1. The center of the WF distribution is around 5.2 eV for 0%- and 25%-IPA films, which then decreases to 4.9 eV as the proportion of IPA increases above 50%. This trend in WF correlates with the cation surface composition, which shows an increase in the Sn content in films obtained from precursors with IPA higher than 50%. With regards to Std. Dev, introducing 25% IPA into the precursor solution leads to significant drop from 115 to 71 meV, which then increases with increasing IPA content. These results are remarkable in the sense that a homogeneous surface electronic landscape and high WF values are obtained in the case of 25%-IPA, which is the same formulation that provided homogeneous elemental composition as extracted from XPS and SIMS analysis. Figure S8 shows 120 μm × 120 μm field of view maps, which confirm the trends observed in Figure 5. Consequently, we have established a clear connection between elemental disorder, surface electronic properties, and formulation of the solution precursor.

### 2.4 Coupling DMF and IPA Solvent Repulsion with CZTSSe Device Performance

**Figure 6:**
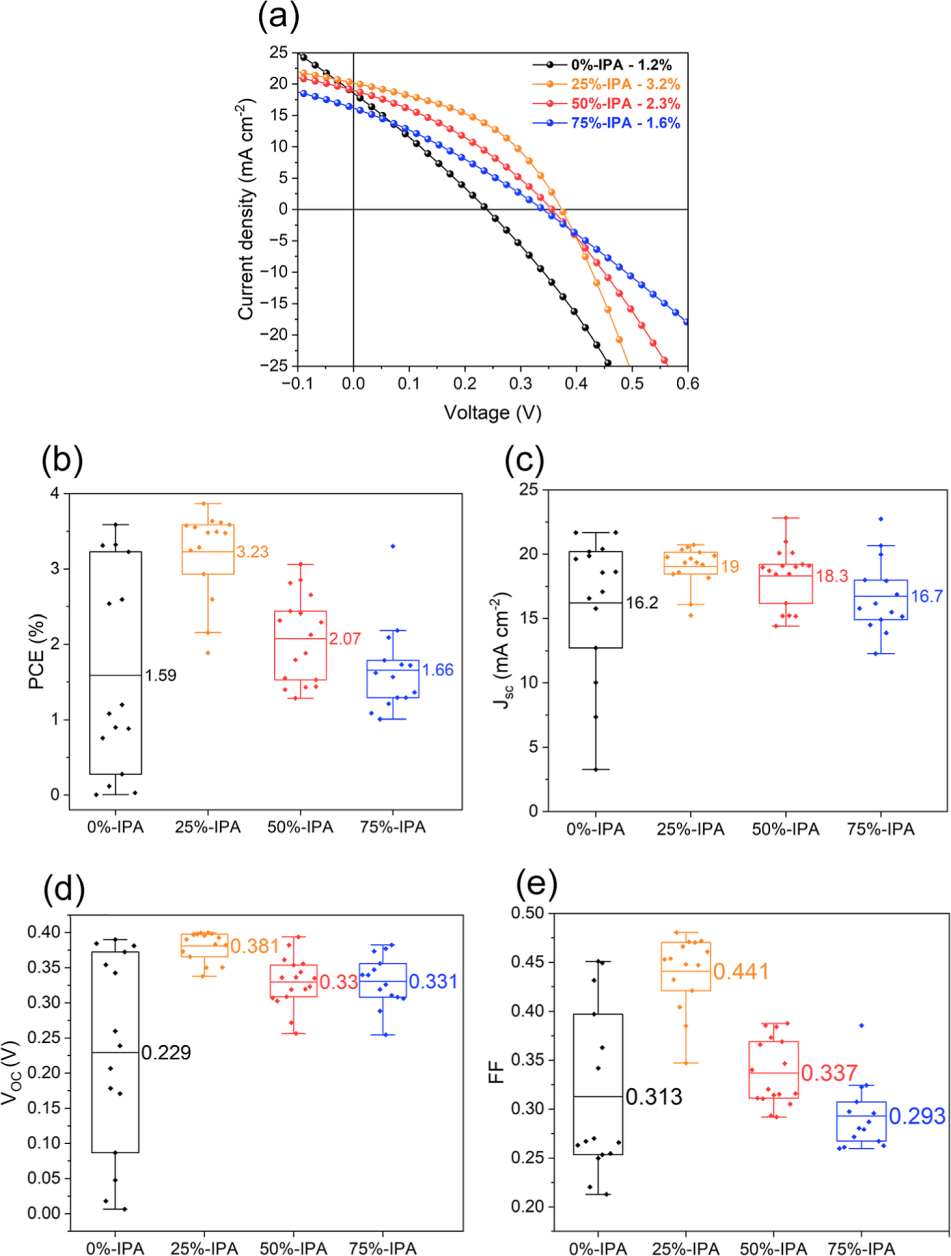
*J*–*V* measurements of representative cells (0.25 cm^2^) processed with various solvent blends (a). Corresponding box plots of power conversion efficiency (PCE) (b), short-circuit current (*J*_sc_) (c), open-circuit voltage (*V*_OC_) (d), and fill factor (FF) (e).

Representative current–voltage curves of CZTSSe absorbers processed with the various solvent composition are illustrated in Figure 6a and Table S3. The device stack consists of glass/FTO/CZTSSe/CdS (50 nm)/i-ZnO (50 nm)/Al:ZnO (500 nm)/Ag (500 nm), as described in the Supporting Information S1. This configuration allows illumination under the substrate (through the Al:ZnO window) or superstrate (through the FTO window) configurations. Under substrate configuration, the champion cell was obtained with 25%-IPA, delivering a power conversion efficiency PCE of 3.87% with a total cell area of 0.25 cm^2^. As shown in Figure S9 and Table S4, the best-performing cell under backward illumination shows efficiencies only below 1%, which indicates significant challenges in the engineering of the back contact.

The key PV metrics extracted from the analysis of current–voltage curves under AM1.5G illumination in the substrate configuration are displayed as box plots in Figure 6b–e (numerical values are summarized in Table S5). We highlight the fact that dispersion in cell metrics is an important parameter to consider when assessing the impact of solvent composition. Consequently, our analysis focuses on the performance of cells delivering PCE close to the mean value for each formulation, rather than that on the champion cells.

The results in Figure 6 show substantial improvement in open-circuit voltage (*V*_OC_) and fill factor (FF) in the case of 25%-IPA, while the mean short-circuit current (*J*_sc_) values are closer for all compositions. A remarkable observation is the high dispersion in *V*_OC_ and FF values observed in 0%-IPA, which is suppressed in 25%-IPA. The resulting large dispersion in PCE can be related to the spatial fluctuation of WF values between 3.5 and 5 eV observed in 0%-IPA (Figures 5a and S8a,b). These results further demonstrate the role of 25% IPA in homogenizing the elemental composition across the absorber layer. Higher IPA content (>50%) leads to a decrease in *V*_OC_ and FF values, which can be linked to Sn segregation to the CZTSSe/CdS junction^55^ and the emergence of Sn secondary phases acting as shunting paths affecting the p–n junction.^54,56,57^ Indeed, Sn disorder has been associated with deep trap states that act as recombination centers, decreasing PCE.^52,58^ Analysis of the EQE spectra depicted in Figure S9b also shows that 25%-IPA has the lowest Urbach energy tails (Figure S9c), which further support the notion that this solvent composition exhibits the lowest elemental disorder.

## 3 Conclusions

Our study reveals, for the first time, the acute impact of precursor solvent mixtures on the morphology, elemental distribution, surface electronic landscape, and PV properties of solution-processed CZTSSe thin films. DMF and IPA solvents show significant repulsion behavior, quantified by an interaction cohesion parameter of 3.06, promoting a nonlinear increase dynamic viscosity, CZTSSe thickness, and surface roughness, with increasing IPA content. Unexpectedly, Sn distribution in the annealed CZTSSe films is also severely affected by the solvent composition, with a decreasing Zn/Sn surface ratio upon increasing the IPA content in the precursor solution. The surface Sn enrichment obtained under high IPA precursor content has a direct influence on the work function distribution, with a mean WF value decreasing from 5.2 to 4.9 eV upon increasing IPA content from 0 to 50%. Throughout our analysis, 25% IPA in the DMF precursor solution leads to high-quality grain growth with a low surface roughness and narrowest WF distribution, which results in boosting the average PCE to 3.23% under substrate illumination on an FTO substrate with a champion of 3.87%.

This study demonstrates the acute importance of solvents in solution processing of semiconductor thin films, which goes beyond rheological properties. In this case, we also demonstrate that the solvent composition can induce changes in the Sn distribution in CZTSSe, which has a significant impact on the optoelectronic properties of the absorber layer.

## Data Availability

Data are available at the University of Bristol data repository, data.bris, at https://doi.org/10.5523/bris.29sxxt06g8m702k3wjdzxo9hl7.

## Supporting Information Available

Experimental details; physical properties of DMF and IPA solvents; image of precursor inks; calibration curve of viscosity; dynamic viscosity of DMF/IPA blends fitted with Lederer–Roegiers Sr. model; Raman shift of O=C–N vibration; fwhm of XRD (112) CZTSSe and Raman A^1^ CZTSe mode; low-magnification SEM images; AFM images; EDX map of the SnSe_2_ phase; SIMS of 25%- and 75%-IPA CZTSSe; XPS of survey spectra and C 1s and O 1s of samples before and after etching; XPS fitting of Sn 3d peak; UPS of primary and secondary edges; EF-PEEM mapping over 120 μm^2^; *J*–*V*, EQE, and Urbach energy analysis of champion solar devices; summary of representative and champion devices; as well as statistical analysis in PV parameters of CZTSSe devices prepared with various proportions of IPA (PDF)
