# Supplementary material for: Utilizing Solvent Repulsion between Dimethylformamide and Isopropanol to Manipulate Sn Distribution for Bifacial Cu2ZnSn(S,Se)4 Solar Cells
Source: ACS Appl Energy Mater. 2024 Nov 27;7(24):11766–74. doi: 10.1021/acsaem.4c01905 (PMC11672233; doi:10.1021/acsaem.4c01905)
Supplement: Supplementary file 1 — ae4c01905_si_001.pdf [file ae4c01905_si_001.pdf]

Supporting Information

Utilising solvent repulsion between dimethylformamide and isopropanol to manipulate Sn distribution for bifacial $Cu_2ZnSn(S,Se)_4$ solar cells

Alice Sheppard,[1,2] Raphael Agbenyeke,[1] Jude Laverock,[1] Laurence King,[1] Jacques Kenyon,[3] Nada Benhaddou,[3] Nicole Fleck,[4] Robert L. Harniman,[1] Andrei Sarua,[2] Devendra Tiwari,[4] Jake W. Bowers,[3] Neil A. Fox,[1,2] David J. Fermin[1*]

[1] School of Chemistry, University of Bristol, Cantocks Close, BS8 1TS Bristol, United Kingdom.
[2] H. H. Wills Physics Laboratory, University of Bristol, Tyndall Avenue, BS8 1TL Bristol, United Kingdom.
[3] Centre for Renewable Energy Systems Technology (CREST), Wolfson School of Mechanical Electrical and Manufacturing Engineering, Loughborough University, Loughborough, LE11 3TU, United Kingdom
[4] Department of Mathematics, Physics and Electrical Engineering, Northumbria University, Ellison Place, NE1 8ST Newcastle upon Tyne, United Kingdom.

Corresponding author: David J. Fermin (david.fermin@bristol.ac.uk)

Table of Contents

S1. Experimental Methods

Table S1. Physical properties of dimethylformamide (DMF) and isopropanol (IPA)

Figure S1: Dynamic viscosity measurements of solvent blends as a function of IPA content

Figure S2: O=C-N bending Raman response of DMF as a function of the IPA content.

Table S2. Broadening of (112) diffraction peak and A^1 Raman mode of CZTSSe absorber films

Figure S3: SEM and AFM images of CZTSSe films processed from precursor solutions with various IPA content.

Figure S4: Secondary ion mass spectroscopy (SIMS) analysis of CZTSSe absorbers obtained from solutions containing 25% (a) and 75% (b) IPA.

Figure S5: XPS spectra of CZTSSe absorber films, before and after 6 mins Ar^+ plasma treatment at 0.5 kV. XPS survey spectra.

Figure S6: Sn 3d XPS spectra of CTZTSSe processed from solution precursors with various IPA content.

Figure S7: Ultraviolet photoelectron spectroscopy of CZTSSe films deposited on FTO employing solution-based precursor with various IPA content

Figure S8: EF-PEEM work function mapping with 120 μm field of view of CZTSSe absorbers

Figure S9: PV device metrics (0.25 cm^2) of CZTSSe absorbers processed with molecular precursors containing different ratio of IPA.

Table S3 Photovoltaic parameters of representative 0.25 cm^2 cells (close to the average cell efficiency) under substrate illumination.

Table S4. Photovoltaic parameters of champion cells under front and back illumination.

Table S5. Mean photovoltaic parameters of substrate illuminated cells

S1. Experimental Methods

Preparation of CZTS precursor solution: CZTS precursor solutions were prepared in a glovebox (Ossila Ltd) at room temperature, with O_2 and H_2O levels of less than 15 ppm. 2.02 mmol $CuCl_2.2H_2O$ (≥ 99.99 %, Sigma Aldrich), 1.22 mmol $SnCl_2$ (≥ 98 %, Sigma Aldrich), 1.47 mmol $ZnCl_2$ (≥ 99.99 %, Sigma Aldrich) and 23.5 mmol thiourea (TU; 98 %, Sigma Aldrich) were sequentially dissolved in 10 mL dimethylformamide (DMF) with the chosen proportion of isopropanol (IPA) (0%, 25%, 50% or 75%) by sonicating at 50 ºC until each salt had dissolved. To maintain a Cu-poor and Zn-rich composition, the molar ratios of Cu/(Sn+Zn), Zn/Sn and TU/metal were 0.75, 1.2 and 5, respectively.

Fabrication of CZTSSe absorber: Commercial FTO-coated soda lime glass (SLG) (Ossila Ltd; 2.5 cm^2; 500 nm; sheet resistance ~ 10 Ωhm/sq) were cleaned by sonication in deionised water (DI), acetone and DI for 10 minutes each, and then dried using Ar flow. Prior to CZTS deposition, the FTO substrates with treated with a UV-O_3 plasma (Jelight UVO-Cleaner Model 42 Series) for 20 minutes. CZTS precursor films were prepared in air via spin coating, by dynamically depositing the precursor solution onto FTO at 500 rpm and then increasing to 2000 rpm for 60 s. Immediately following this, the wet film was annealed on a hotplate at 350 ºC for 2 minutes and allowed to cool naturally. This coating-annealing-cooling cycle was repeated 13 times. For selenisation, the CZTS film was placed into a graphite box, with 28 sccm flow of argon (1 atm), with 600 mg of Se powder and heated to 530 ºC for 20 minutes in a rapid thermal annealing (RTA) furnace (MTI OTF-1200X). Upon the completion of the selenisation process, the furnace was allowed to cool naturally to 50 ºC before the removal of the CZTSSe film.

Device Fabrication: CZTSSe solar devices on FTO were completed by depositing 50 nm of CdS buffer layer using chemical bath deposition (CBD) from an aqueous bath containing $CdSO_4$, TU and ammonium hydroxide, at a temperature beginning at 58 ºC and increasing to 70 ºC by the end of the deposition. Following this, 50 nm of i-ZnO and 500 nm Al-doped ZnO (AZO) were deposited by radio frequency (RF)-sputtering. Ag (500 nm) top contacts were fabricated by thermal evaporation and solar cells, at an area of 0.25 cm^2, were defined by mechanical scribing. Based on the DMF:IPA solvent ratio of the precursor solutions, the solar cell devices are referred to as 0%-IPA, 25%-IPA, 50%-IPA and 75%-IPA in this study.

Film Characterisation: Dynamic viscosity of DMF and IPA solvent blends, with and without the CZTS precursor salts, were measured in a 10 mL U-tube Reverse Flow viscometer at 25 °C, where the viscometer was calibrated using 4 known solvents and the measurement was repeated 5 times. Error determination of dynamic viscosity was determined using partial differentiation. Raman analysis of DMF and IPA solutions was measured at 785 nm (Perkin Elmer RamanFlex 400) with a polystyrene reference, with a 3 × 10 s exposure. Thin film Raman spectra (Renishaw inVia) were acquired using a 532 nm laser excitation source focused into about 1 µm spot using high NA microscope lens. X-ray diffraction (XRD) patterns (2θ = 10-60º, 0.02º, 1 second, 60 rpm) were collected by a Bruker D8 Advance with a PSD LynxEye X-ray powder diffraction system using Cu Kα (λ = 1.54184Å) X-ray source. The angle of (112) peak can be used to estimate the S/(S+Se) chalcogen ratio by analysing the difference from the literature (112) values from the pure sulfide phase (28.45° - JCPDS 04-015-0223) and pure selenide phase (27.11° - JCPDS 00-066-0216). All spectra were calibrated to FTO (110) peak at 26.7°. Top-down and cross-sectional morphology were

measured using scanning electron microscopy (SEM) (Jeol IT300 SEM). Atomic force microscopy (AFM) (TUNA Bruker multimode VIII with Nanoscope V controller and PeakForce feedback) using Pt/Ir coated tip with nominal radius of 20 nm and applying 500 mV bias. Secondary ion mass spectroscopy (SIMS) was measured with a Hidden Analytical gas ion gun and quadrupole detector operating an Ar^+ beam at 4 keV, raster area of 500 × 500 μm^2 and a grating of 10%. Ultra-high vacuum (UHV) X-ray photoelectron spectroscopy (XPS), energy-filtered photoemission electron microscopy (EF-PEEM) and ultraviolet photoelectron spectroscopy (UPS) analysis were performed at the Bristol Ultraquiet NanoESCA Laboratory using a NanoESCA II (ScientaOmicron/FOCUS) EF-PEEM instrument and an Argus (ScientaOmicron) XPS analyser. For UHV sample preparation, bare CZTSSe absorbers were etched with 0.5 kV Ar^+ plasma at 45º for 6 minutes and at a pressure of 1.2×10^{-5} mbar with a total sputter flux of approximately 130 µA min. XPS spectra pre- and post-etching were measured using a monochromatic Al Kα source (1486.7 eV) with pass energies of 100 and 50 eV for survey and high-resolution spectra, respectively, the latter corresponding to an energy resolution of 500 meV at full-width-half-maximum. The energy axis was referenced to the Fermi level of sputtered polycrystalline silver, and all samples did not show any signs of charging during the measurement. The intensity scale was calibrated to parameterised spectra of low-density polyethylene (LDPE).[56, 57] Quantification of metal ratios were performed using Cu $2p_{3/2}$ at 932 eV, Zn $2p_{3/2}$ at 1022 eV and Sn $3d_{5/2}$ at 486 eV. EF-PEEM and UPS were performed under UHV conditions ($\approx 4\times10^{-11}$ mbar) using monochromatic He I (21.2 eV) as the excitation source. A contrast aperture was inserted into the back focal plane of the microscope for work function maps to improve the lateral resolution, and the energy resolution of the analyser was set to 100 meV at a pass energy of 50 eV. UPS data was measured between binding energies of 19 and -1 eV.

Device Characterisation: Photocurrent density-voltage (J-V) characteristics were measured under AM1.5G conditions using a solar simulator (Wavelabs Sinus-70 light) with an integrated power density of 100 mW cm^{-2}. The external quantum efficiency (EQE) spectra were obtained at 0 V bias with 5 nm spectral resolution using a Bentham PVE300 system, with a monochromator (Bentham TMc300), a dual halogen and single xenon as light sources, and transformer (x500 474 type pre-amp) under half sun conditions. Short circuit current density (J_{sc}) from EQE was measured by integrating over the wavelength range of 300 – 1000 nm.

Table S1. Physical properties of dimethylformamide (DMF) and isopropanol (IPA), as reported in Merck SDS 68-12-2 and 67-63-0.

Solvent	Dynamic Viscosity at 20 °C (mPa.s)	Vapour Pressure at 20 °C (hPa)	Surface tension (mN/m)	Density at 25 °C (g/cm^3)	Boiling Point (°C)
DMF	0.86	3.77	37.1	0.944	153
IPA	2.2	43	23.0	0.785	82

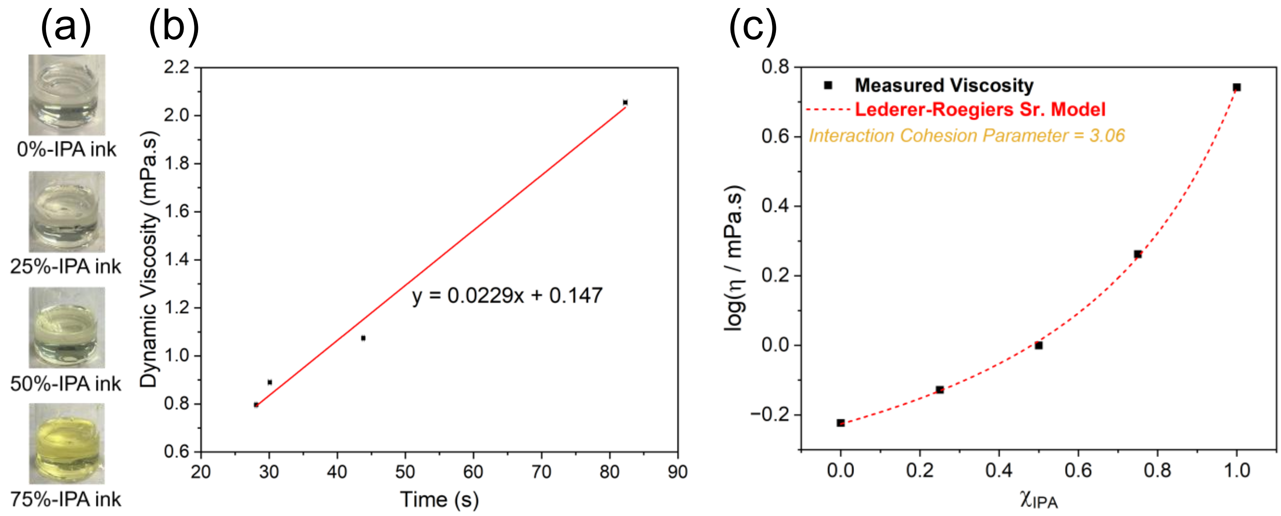

Figure S1: Image of molecular precursor inks with various IPA content (a). Calibration curve for viscosity measurements, using the known viscosities of DMF, water, ethanol, and isopropanol as references (b), and dynamic viscosity (η) curve for binary DMF:IPA blending (without precursor salts), fitted with Lederer-Roegiers Sr. Model, with a cohesion parameter of 3.06 (c).

Lederer-Roegiers Sr. Model (Equation S1)[39-41]:

$$\ln \eta_{12} = \ln \eta_1 + \frac{\alpha x_2}{x_1 + \alpha x_2}(\ln \eta_2 - \ln \eta_1) \quad \text{(eq. S1)}$$

Where η_{12}, η_1 and η_2 are the dynamic viscosity's of the solvent blend, and pure solvents 1 and 2 in mPa.s, respectively, α is the interaction cohesion parameter, and x_1 and x_2 are the mole fractions of solvents 1 and 2, respectively.

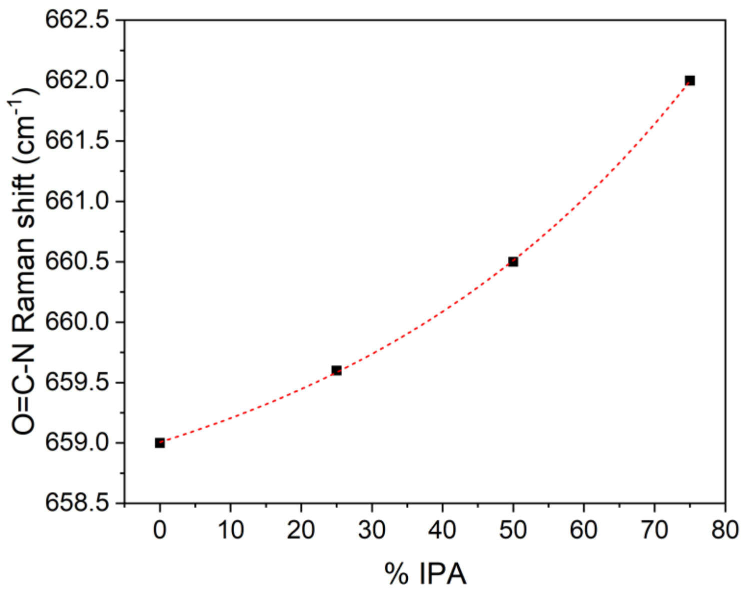

Figure S2: Non-linear shift of the Raman responses associated with O=C-N bending vibration in DMF as a function of the IPA content in the solvent blend.

Table S2. Broadening of (112) diffraction peak and A^1 Raman mode of CZTSSe absorber films. Error in the fullwidth half maximum (FWHM) of the XRD and Raman features is ±0.02° and 1 cm^{-1}, respectively.

% IPA	(112) FWHM (°)	CZTSe A^1 FWHM (cm^{-1})
0	0.25	14
25	0.14	8
50	0.16	10
75	0.23	13

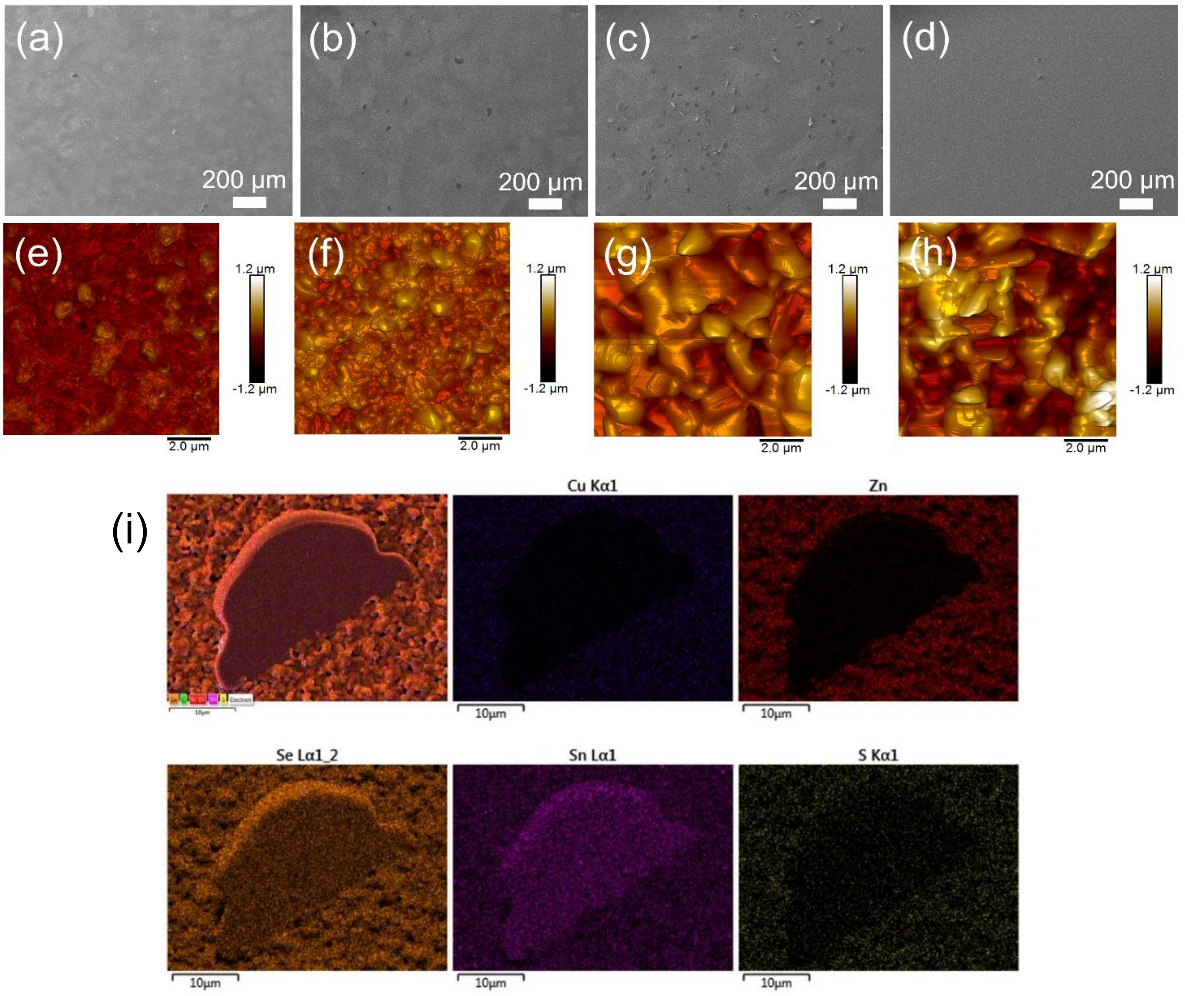

Figure S3: Top-down SEM images at 75 x (a-d) and 10 µm × 10 µm AFM image (e-h) of 0%- (a,e) 25%- (b,f), 50%- (c,g) and 75%-IPA (d,h) CZTSSe absorbers**.** EDX map of $SnSe_2$ secondary phase on 50%-IPA sample (i).

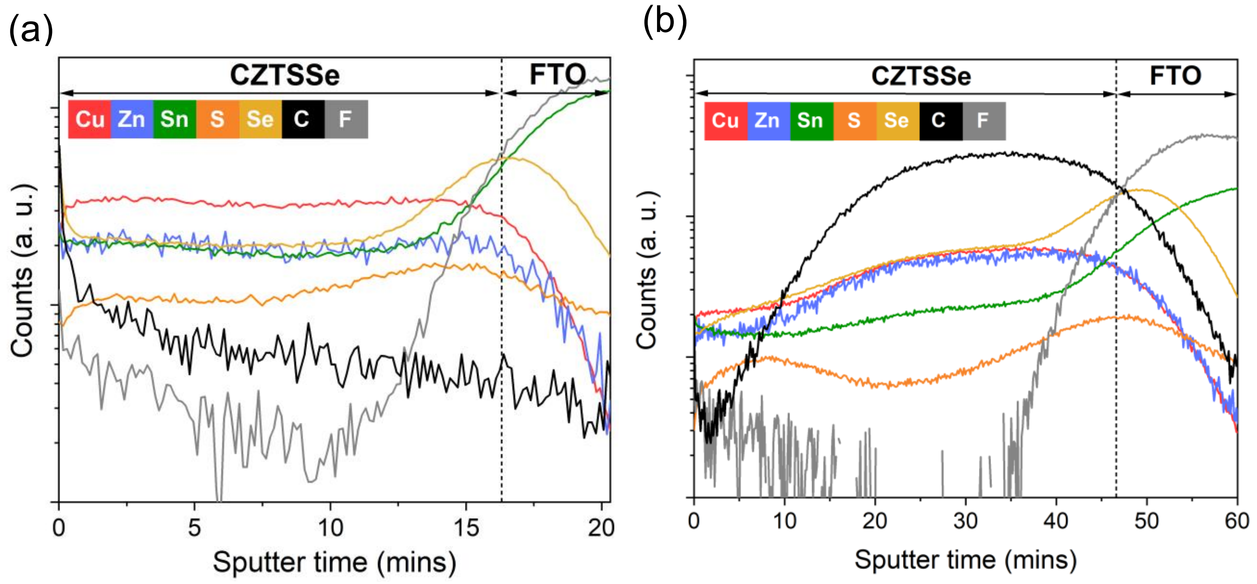

Figure S4: Secondary ion mass spectroscopy (SIMS) analysis of 25%- (a) and 75%-IPA (b) CZTSSe absorbers.

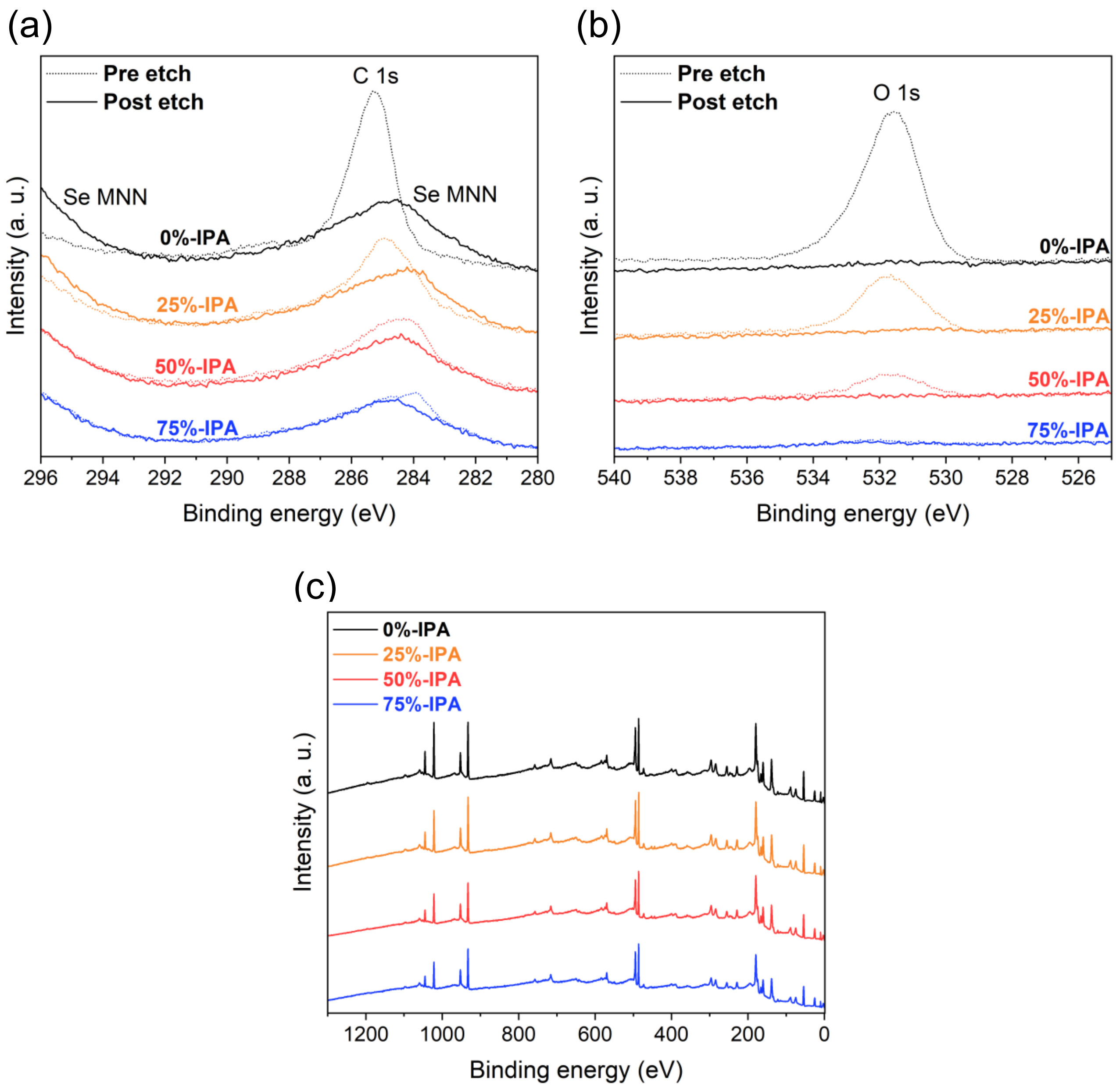

Figure S5: XPS spectra of C 1s (a) and O 1s (b) of CZTSSe absorber films, before and after 6 mins Ar^+ plasma treatment at 0.5 kV. XPS survey spectra (c) of the CZTSSe films processed from the various solvent blends after surface pretreatment.

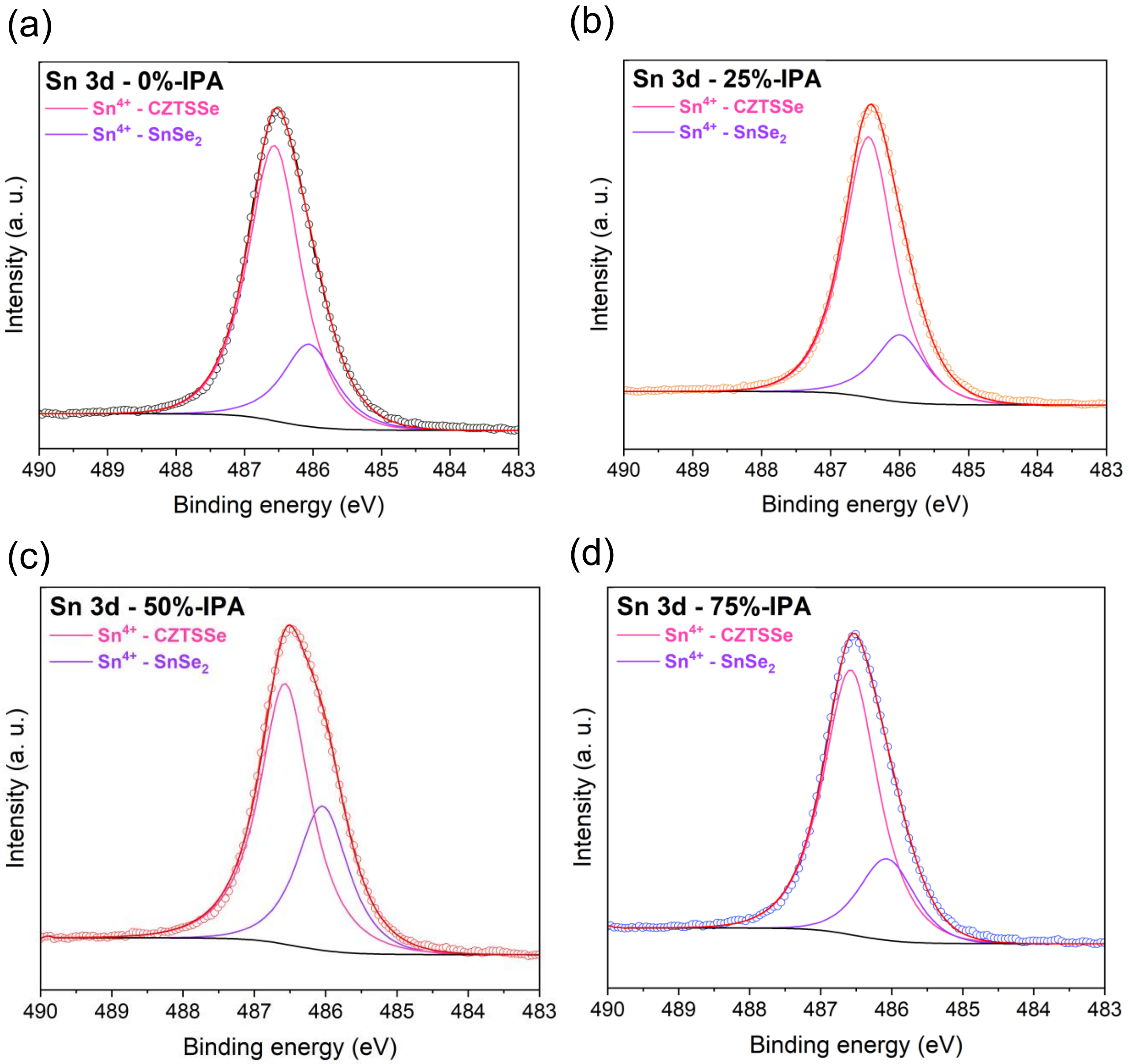

Figure S6: Sn 3d XPS spectra of CTZTSSe processed from solution precursors containing 0 (a), 25 (b), 50 (c) and 75%-IPA (d). Two Sn environments are fitted corresponding to the main CZTSSe phase (pink) and a $SnSe_2$ secondary phase (purple).

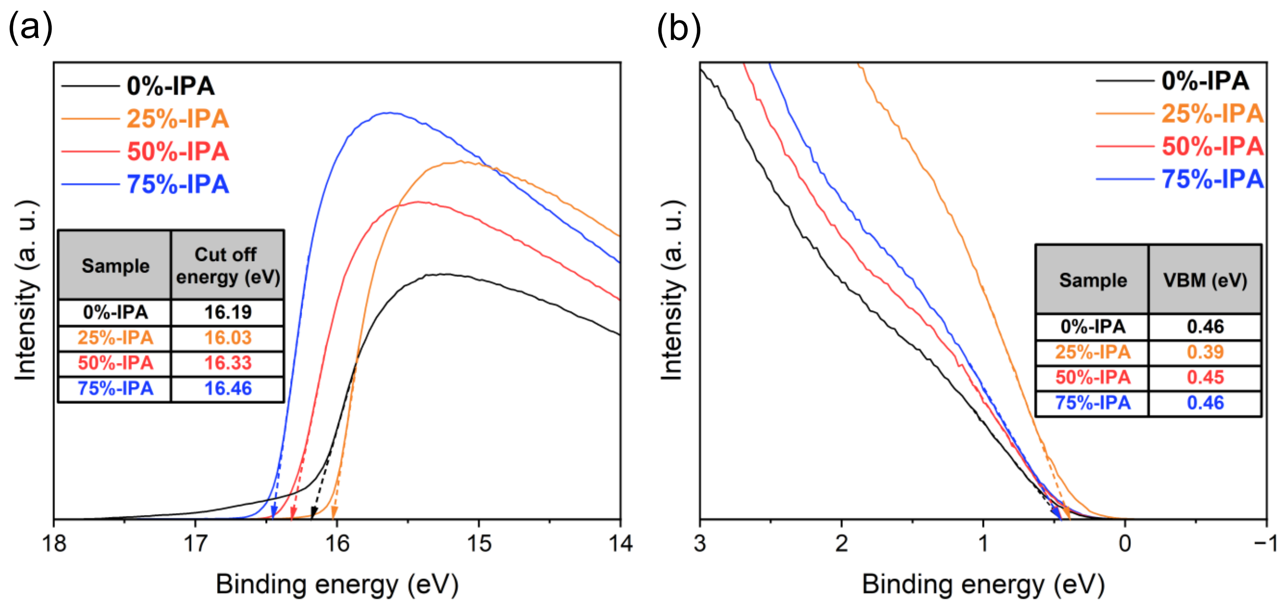

Figure S7: Ultraviolet photoelectron spectroscopy of CZTSSe films deposited on FTO employing solution-based precursor with various IPA content: secondary cutoff energy (a) and valence band region (b).

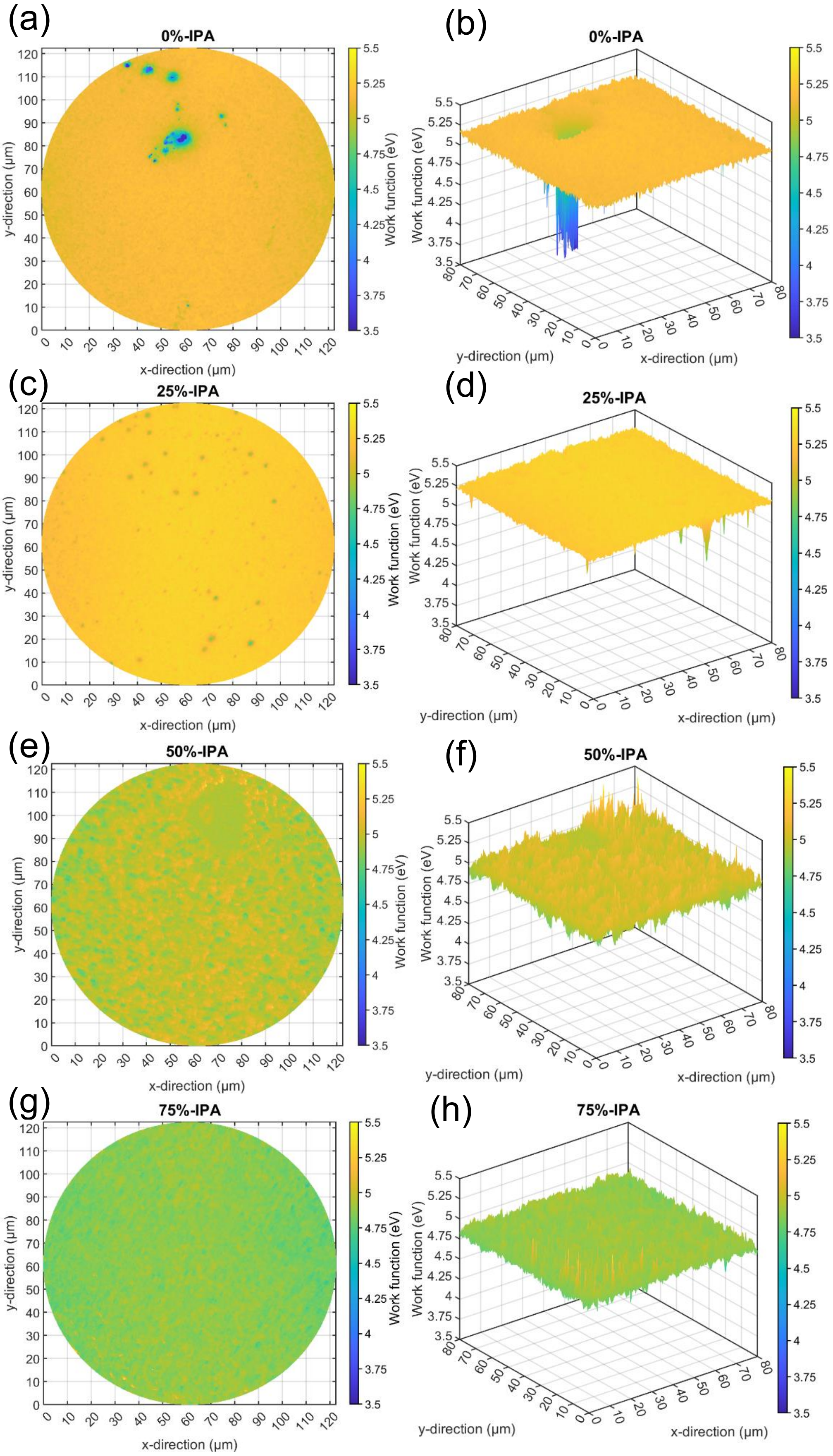

Figure S8: EF-PEEM work function mapping with 120 μm field of view of CZTSSe absorbers processed with 0% (a,b), 25% (c,d), 50% (e,f) and 75% IPA (g,h).

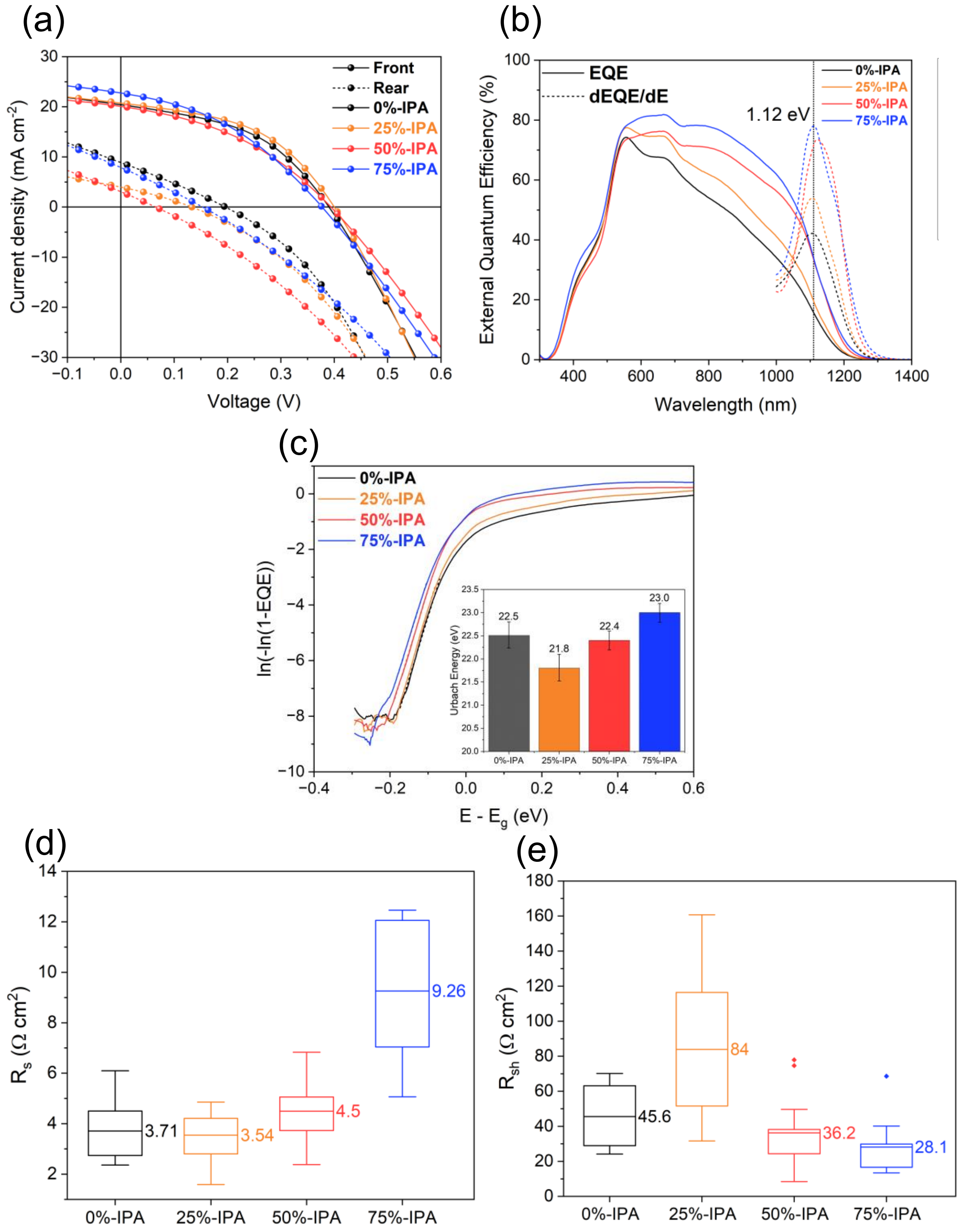

Figure S9: PV device metrics (0.25 cm^2) of CZTSSe absorbers processed with molecular precursors containing different ratio of IPA. J-V curves under substrate and superstrate illumination (a); external quantum efficiency (EQE) of champion cells (b); Urbach energy estimated from EQE spectra (c); box plots of device series (d) and shunt (e) resistances under AM 1.5 illumination.

Table S3 Photovoltaic parameters of representative 0.25 cm^2 cells (close to the average cell efficiency) under substrate illumination.

% IPA	PCE (%)	J_{sc} (mA cm^{-2})	V_{OC} (V)	FF (%)
0	1.20	18.6	0.239	27.0
25	3.25	20.1	0.373	43.2
50	2.32	19.0	0.356	34.0
75	1.62	16.2	0.339	29.6

Table S4. Photovoltaic parameters of champion cells under front (F) and back (B) illumination. Short-circuit current obtained from integration of the corresponding EQE spectrum (J_{int}), percentage difference with respect to *J*-*V* curves ($J_{SC,error}$) and ideality factor (*n*).

Illumination	IPA %	PCE (%)	J_{sc} (mA cm^{-2})	V_{OC} (V)	FF (%)	$J_{SC,int}$ (mA cm^{-2})	$J_{SC,error}$ (%)	*n*
F	0	3.59	20.4	0.390	45.1	20.5	1	1.79
B	0	0.47	8.9	0.196	27.1	-	-	-
F	25	3.87	20.7	0.400	46.6	22.4	8	2.04
B	25	0.15	4.0	0.133	29.3	-	-	-
F	50	3.06	20.1	0.394	38.8	23.8	18	2.84
B	50	0.05	3.0	0.065	25.8	-	-	-
F	75	3.30	22.7	0.377	38.5	26.2	15	2.45
B	75	0.32	7.9	0.153	26.5	-	-	-

Table S5. Mean photovoltaic parameters of substrate illuminated cells.

% IPA	PCE (%)	J_{sc} (mA cm^{-2})	V_{OC} (V)	FF (%)	R_s (Ω cm^2)	R_{sh} (Ω cm^2)
0	1.59±1.35	16.2±5.5	0.229±0.141	31.0±8.4	3.71±1.31	45.6±19.4
25	3.23±0.58	19.0±1.6	0.381±0.020	44.0±3.7	3.54±0.88	84.0±42.0
50	2.07±0.58	18.3±2.0	0.330±0.037	34.0±3.4	4.50±1.17	36.2±17.3
75	1.66±0.59	16.7±2.9	0.331±0.036	29.0±3.4	9.26±2.80	28.1±14.2
